# Development and validation of machine learning model to predict early death of melanoma brain metastasis patients

**DOI:** 10.3389/fonc.2025.1517961

**Published:** 2025-07-08

**Authors:** Maierdanjiang Maihemuti, Maiheliya Kamaierjiang, Aierpati Maimaiti, Junshen Wu, Zhibing Dai, Renbing Jiang

**Affiliations:** ^1^ Department of Bone and Soft Tissue, Affiliated Tumor Hospital of Xinjiang Medical University, Xinjiang, China; ^2^ Department of Cardiovascular Medicine, General Hospital of Xinjiang Military Region, Urumqi, Xinjiang, China; ^3^ Department of Neurosurgery, Neurosurgery Centre, The First Affiliated Hospital of Xinjiang Medical University, Urumqi, Xinjiang, China

**Keywords:** melanoma, brain metastasis, early death, machine learning, prognosis

## Abstract

**Background:**

Melanoma has the third highest rate of brain metastases among all cancers and is associated with poor long-term survival. This study aimed to develop machine learning models to predict early death in melanoma brain metastasis (MBM) patients to guide clinical decision-making

**Methods:**

We analyzed MBM patients from the SEER database and Xinjiang Medical University. Patients were randomly divided into training and testing cohorts (7:3 ratio). Seven machine learning models were developed and validated using cross-validation, ROC analysis, decision curve analysis, and calibration curves to predict cancer-specific early death (CSED) and all-cause early death (ACED) within 3 months of diagnosis.

**Results:**

Among 1,547 MBM patients, 531 (34.3%) experienced CSED, and 554 (35.8%) experienced ACED. Key predictive factors included age, treatment modalities (radiation, chemotherapy, surgery), tumor characteristics (ulceration), and extracranial metastases (bone, liver). XGBoost achieved the best performance for ACED prediction (AUC=0.776), while logistic regression performed best for CSED prediction (AUC=0.694). External validation confirmed model reliability with comparable performance.

**Conclusion:**

These machine learning models demonstrate strong predictive performance and may assist clinicians in early risk stratification and treatment planning for MBM patients. The models provide objective risk assessment tools that could improve patient counseling and guide aggressive versus palliative care decisions.

## 1 Introduction

Melanoma is the most lethal form of skin cancer, known for its aggressive spread to multiple organs (1, 2). Malignant melanoma ranks third in brain metastasis incidence among all cancers, followed by lung and breast cancer (3). Central nervous system involvement is evident in 40-60% of advanced melanoma patients (4, 5), and is the direct cause of death in 60-70% of melanoma patients (1). The long-term survival rate of MBM patients has been under 10% (6). Without treatment, MBM advances quickly, with a 5-year survival rate of less than 10%, and most patients succumb within 7.5 months (7). According to previous studies across multiple cancer types, early death (ED) was defined as patient death within 3 months of the first diagnosis (8, 9), a definition that has been consistently validated in gastric cancer, lung cancer with brain metastasis, and specifically in melanoma populations (10, 11), demonstrating its clinical relevance across diverse cancer types and treatment eras. More than 95% of early melanoma deaths were metastatic melanoma and resulted in cancer-specific death (12). Previous studies have reported factors influencing the incidence and prognosis of MBM, but the conclusions remain disputed (12–14). Therefore, it’s necessary to recognize the risk factors associated with ED from the large sample data and develop models to assess the likelihood of early death in MBM patients accurately.

Retrospective studies using historical data play a crucial role in clinical decision-making (15). Artificial intelligence has been widely utilized in both cardiovascular disease (16) and oncology (17) to develop predictive models. Machine learning (ML), a branch of artificial intelligence, can automatically analyze large datasets, identify hidden relationships between factors and survival outcome, and build highly effective models to predict outcomes for new data. This study aimed to retrospectively analyze the clinicopathological characteristics associated with ED and build ML models to predict the ED of MBM patients. The predictive ML models may assist patients and doctors in making optimal clinical decisions.

## 2 Materials and methods

### 2.1 Data collection

The SEER*Stat software (www.seer.cancer.gov, version 8.43)was used to extract data on MBM patients from the SEER database. The International Classification of Oncology Diseases and Version 3 (ICD-O-3) criteria were used to determine the primary cancer site, and the diagnosis was finally histologically confirmed. CSED was defined as patients dying due to MBM within three months. ACED was defined as patients dying due to all causes within three months. For the SEER database component, ethics approval was not required as SEER data is publicly available and de-identified.

The inclusion criteria were as follows: (1) primary cancer only, (2) patients histologically confirmed with MBM from 2010 to 2020 in the SEER database. The exclusion criteria were as follows: (1) diagnosis confirmed by autopsy or death certificate only, (2) age less than 18 years, (3) with unknown clinicopathological information, (4) Unknown metastatic site. For metastatic site identification, we utilized the Collaborative Stage (CS) metastatic site codes in the SEER database to identify patients with brain metastasis and to determine the presence of other distant metastases, including liver, lung, and bone. It’s important to note that the SEER database records these metastases as binary variables (Yes/No) without providing further anatomical subdivision. This limitation prevented more granular analyses of metastatic patterns in our study.

Regarding missing data handling, we implemented a conservative approach to maintain data integrity. For categorical variables where “Unknown” was explicitly coded as a category in the SEER database (such as ulceration status, marital status, etc.), we retained these patients and analyzed “Unknown” as a separate category rather than excluding them. This approach is consistent with previous SEER-based studies and preserves the sample size while acknowledging data limitations transparently (18). This strategy allowed us to develop more robust machine learning models that can provide predictions even when certain data points might be missing in clinical practice.

**Figure 1:**
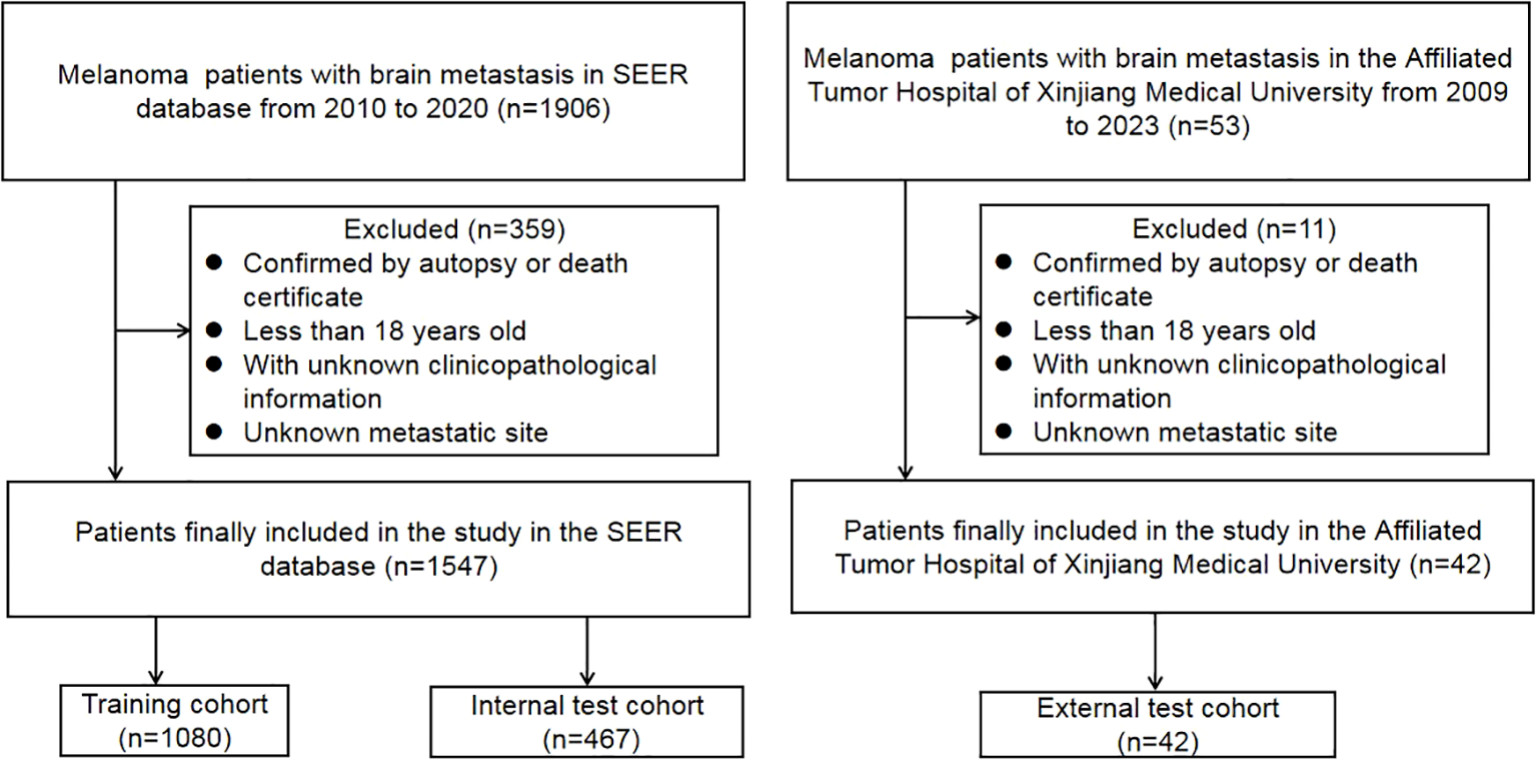
Flowchart of the study process.

To confirm the reliability of the data, the included patients from the SEER database were randomly divided into training and testing cohorts in a ratio of 7:3. The validation data cohort was extracted from the Affiliated Tumor Hospital of Xinjiang Medical University from 2010 to 2023, and follow-up lasted until October 31, 2023(**Figure 1**). Approval for this study was obtained from the Institutional Review Board of the Affiliated Tumor Hospital of Xinjiang Medical University.

### 2.2 Study design

Independent risk factors were identified through a systematic variable selection process using univariate and multivariate logistic regression analysis. Initially, all candidate variables were evaluated using univariate logistic regression. Features with p values <0.05 in the univariate analysis were then entered into a backward stepwise multivariate logistic regression model. The backward elimination process started with all significant univariate variables included in the full model and sequentially removed the variable with the highest p-value (least significant) at each step, provided that p>0.10. This process continued iteratively until all remaining variables in the model achieved statistical significance (p<0.05). The final multivariate model retained only variables that remained statistically significant after controlling for other factors through this backward elimination process. These independently significant risk factors identified through backward stepwise regression were then used as input features to construct the machine learning models. Seven ML models were developed, including logistic regression (LR), K-nearest neighbor (KNN), support vector machine (SVM), decision tree (DT), random forest (RF), XGBoost, and LightGBM. The training cohort was used for building ML models and cross-validation, while testing and validation cohorts were used to evaluate the predictive performance of the models.

### 2.3 Statistical analysis

Categorical variables are presented as numbers (%), and comparisons were made using the Pearson Chi-square test. Univariate and multivariate logistic regression analyses were conducted to determine risk factors. All statistical analysis, model development, and validation are implemented in R software (version 4.3.0). A two-sided p-value <0.05 was considered statistically significant.

Cross-validation methodology: All models were evaluated using stratified 10-fold cross-validation to ensure robust performance estimation while maintaining outcome distribution balance across folds. For the logistic regression model, which requires no hyperparameter tuning, cross-validation was used solely for unbiased performance evaluation. For the remaining six models (KNN, SVM, DT, RF, XGBoost, and LightGBM), a nested cross-validation approach was implemented where hyperparameter optimization occurred independently within each fold to prevent data leakage and ensure valid generalizability estimates.

Hyperparameter optimization: Each model’s hyperparameters were tuned using random grid search within the cross-validation framework. KNN optimized the number of neighbors (3-11); SVM tuned cost (-5 to 5) and RBF sigma (-4 to -1); DT optimized tree depth (3-7), minimum node size (5-10), and cost complexity (-6 to -1); RF tuned the number of variables per split (2-10), number of trees (200-500), and minimum node size (20-50); XGBoost optimized six parameters including learning rate, tree depth, and regularization terms; LightGBM similarly tuned six key parameters. For models requiring data preprocessing (KNN, SVM), normalization and dummy coding were performed within each fold using the tidymodels recipe framework to prevent data leakage.

Model validation strategy: Model performance was evaluated through a three-tier validation approach: (1) Internal validation using 10-fold cross-validation on the training cohort to assess model stability and select optimal hyperparameters; (2) Temporal validation using an internal test set (30% holdout from the same institution/database) that was never used during model development or cross-validation; and (3) External validation using an independent cohort from a different institution/database/time period to evaluate model generalizability across different populations and clinical settings. This comprehensive validation strategy ensures robust assessment of model performance and clinical applicability across diverse patient populations.

ROC analysis, calibration curves, and DCA curves were used to evaluate the models’ accuracy.

## 3 Results

### 3.1 Patient characteristics

A total of 1547 MBM patients from the SEER database were finally included in this study. Among them, 531 (34.3%) cases suffered from CSED, and 554 (35.8%) cases suffered from ACED. The details of the patients are displayed in **Table 1**. For ACED, most patients were > 60 years old (50%), male (74.2%), white (98%), did not receive surgery (89%), received radiation (75.3%), and did not receive or had unknown chemotherapy status (83.2%). As for CSED, most of the patients > 60 years old (49.5%), male (74%), white (97.9%), without surgery (88.7%), with radiation (75.9%), without or unknown whether perform chemotherapy (82.9%). The demography of patients in the training cohort (n=1080), test cohort (n=467), and validation (n=42) cohort is shown in **Table 2**.

**Table 1:** Baseline characteristics of the BMM patients from the SEER database.

Variables		ALL (N=1547)	No early death (N=993)	All cause early death(N=554)	No cancer-specific early death (N=1016)	Cancer-specific early death (N=531)
Age	<40	110 (7.1%)	81 (8.2%)	29 (5.2%)	82 (8.1%)	28 (5.3%)
	40-65	816 (52.7%)	568 (57.2%)	248 (44.8%)	576 (56.7%)	240 (45.2%)
	>65	621 (40.1%)	344 (34.6%)	277 (50%)	358 (35.2%)	263 (49.5%)
Sex	Male	1118 (72.3%)	707 (71.2%)	411 (74.2%)	725 (71.4%)	393 (74%)
	Female	429 (27.7%)	286 (28.8%)	143 (25.8%)	291 (28.6%)	138 (26%)
Race	White	1512 (97.7%)	969 (97.6%)	543 (98%)	992 (97.6%)	520 (97.9%)
	Black	11 (0.7%)	7 (0.7%)	4 (0.7%)	7 (0.7%)	4 (0.8%)
	Other	24 (1.6%)	17 (1.7%)	7 (1.3%)	17 (1.7%)	7 (1.3%)
Marital	Married	888 (57.4%)	587 (59.1%)	301 (54.3%)	597 (58.8%)	291 (54.8%)
	Unmarried	659 (42.6%)	406 (40.9%)	253 (45.7%)	419 (41.2%)	240 (45.2%)
Primary Site	Skin, NOS	1217 (78.7%)	769 (77.4%)	448 (80.9%)	788 (77.6%)	429 (80.8%)
	Skin other/unspecific parts of face	18 (1.2%)	12 (1.2%)	6 (1.1%)	13 (1.3%)	5 (0.9%)
	Skin of scalp and neck	42 (2.7%)	31 (3.1%)	11 (2%)	31 (3.1%)	11 (2.1%)
	Skin of trunk	116 (7.5%)	77 (7.8%)	39 (7%)	78 (7.7%)	38 (7.2%)
	Skin of upper limb and shoulder	58 (3.7%)	36 (3.6%)	22 (4%)	36 (3.5%)	22 (4.1%)
	Skin of lower limb and hip	57 (3.7%)	40 (4%)	17 (3.1%)	40 (3.9%)	17 (3.2%)
	Choroid	2 (0.1%)	2 (0.2%)	0 (0%)	2 (0.2%)	0 (0%)
	External ear	3 (0.2%)	3 (0.3%)	0 (0%)	3 (0.3%)	0 (0%)
	Other rare sites	34 (2.2%)	23 (2.3%)	11 (2%)	25 (2.5%)	9 (1.7%)
Surgery	No	1342 (86.7%)	849 (85.5%)	493 (89%)	871 (85.7%)	471 (88.7%)
	Yes	205 (13.3%)	144 (14.5%)	61 (11%)	145 (14.3%)	60 (11.3%)
Radiation	No/Unknown	323 (20.9%)	186 (18.7%)	137 (24.7%)	195 (19.2%)	128 (24.1%)
	Yes	1224 (79.1%)	807 (81.3%)	417 (75.3%)	821 (80.8%)	403 (75.9%)
Chemotherapy	No/Unknown	1099 (71%)	638 (64.2%)	461 (83.2%)	659 (64.9%)	440 (82.9%)
	Yes	448 (29%)	355 (35.8%)	93 (16.8%)	357 (35.1%)	91 (17.1%)
Ulceration	No Ulceration	283 (18.3%)	208 (20.9%)	75 (13.5%)	213 (21%)	70 (13.2%)
	Uleration	79 (5.1%)	47 (4.7%)	32 (5.8%)	47 (4.6%)	32 (6%)
	Unknown	1185 (76.6%)	738 (74.3%)	447 (80.7%)	756 (74.4%)	429 (80.8%)
Breslow depth	<=1	675 (43.6%)	450 (45.3%)	225 (40.6%)	459 (45.2%)	216 (40.7%)
	1.01-2	22 (1.4%)	17 (1.7%)	5 (0.9%)	17 (1.7%)	5 (0.9%)
	2.01-4	26 (1.7%)	17 (1.7%)	9 (1.6%)	17 (1.7%)	9 (1.7%)
	>4	55 (3.6%)	34 (3.4%)	21 (3.8%)	34 (3.3%)	21 (4%)
	Unknown	769 (49.7%)	475 (47.8%)	294 (53.1%)	489 (48.1%)	280 (52.7%)
Bone metastasis	No	1211 (78.3%)	818 (82.4%)	393 (70.9%)	833 (82%)	378 (71.2%)
	Yes	336 (21.7%)	175 (17.6%)	161 (29.1%)	183 (18%)	153 (28.8%)
Liver metastasis	No	1190 (76.9%)	803 (80.9%)	387 (69.9%)	820 (80.7%)	370 (69.7%)
	Yes	357 (23.1%)	190 (19.1%)	167 (30.1%)	196 (19.3%)	161 (30.3%)
Lung metastasis	No	700 (45.2%)	477 (48%)	223 (40.3%)	492 (48.4%)	208 (39.2%)
	Yes	847 (54.8%)	516 (52%)	331 (59.7%)	524 (51.6%)	323 (60.8%)
Months from diagnosis to therapy	0	952 (61.5%)	588 (59.2%)	364 (65.7%)	604 (59.4%)	348 (65.5%)
	>=1	595 (38.5%)	405 (40.8%)	190 (34.3%)	412 (40.6%)	183 (34.5%)
Media household income	<$45,000	88 (5.7%)	53 (5.3%)	35 (6.3%)	54 (5.3%)	34 (6.4%)
	$45,000–$59,999	283 (18.3%)	176 (17.7%)	107 (19.3%)	178 (17.5%)	105 (19.8%)
	$60,000–$74,999	517 (33.4%)	335 (33.7%)	182 (32.9%)	342 (33.7%)	175 (33%)
	>$74,999	659 (42.6%)	429 (43.2%)	230 (41.5%)	442 (43.5%)	217 (40.9%)

**Table 2:** Baseline characteristics of the training, testing, and validation cohort patients.

Variables		Train (N=1080)	Test (N=467)	Validation (N=42)
Age	<40	79 (7.3%)	31 (6.6%)	2 (4.8%)
	40-65	583 (54%)	233 (49.9%)	26 (61.9%)
	>65	418 (38.7%)	203 (43.5%)	14 (33.3%)
Sex	Male	786 (72.8%)	332 (71.1%)	33 (78.6%)
	Female	294 (27.2%)	135 (28.9%)	9 (21.4%)
Race	White	1060 (98.1%)	452 (96.8%)	NA
	Black	5 (0.5%)	6 (1.3%)	NA
	Other	15 (1.4%)	9 (1.9%)	NA
Marital	Married	621 (57.5%)	267 (57.2%)	15 (35.7%)
	Unmarried	459 (42.5%)	200 (42.8%)	27 (64.3%)
Primary Site	Skin, NOS	846 (78.3%)	371 (79.4%)	31 (73.8%)
	Skin other/unspecific parts of face	14 (1.3%)	4 (0.9%)	NA
	Skin of scalp and neck	30 (2.8%)	12 (2.6%)	NA
	Skin of trunk	88 (8.1%)	28 (6%)	5 (11.9%)
	Skin of upper limb and shoulder	41 (3.8%)	17 (3.6%)	NA
	Skin of lower limb and hip	33 (3.1%)	24 (5.1%)	3 (7.1%)
	Choroid	1 (0.1%)	1 (0.2%)	NA
	External ear	3 (0.3%)	0 (0%)	1 (2.4%)
	Other rare sites	24 (2.2%)	10 (2.1%)	2 (4.8%)
Surgery	No	932 (86.3%)	410 (87.8%)	36 (85.7%)
	Yes	148 (13.7%)	57 (12.2%)	6 (14.3%)
Radiation	No/Unknown	222 (20.6%)	101 (21.6%)	14 (33.3%)
	Yes	858 (79.4%)	366 (78.4%)	28 (66.7%)
Chemotherapy	No/Unknown	768 (71.1%)	331 (70.9%)	30 (71.4%)
	Yes	312 (28.9%)	136 (29.1%)	12 (28.6%)
Ulceration	No Ulceration	199 (18.4%)	84 (18%)	6 (14.3%)
	Ulceration	56 (5.2%)	23 (4.9%)	2 (4.8%)
	Unknown	825 (76.4%)	360 (77.1%)	34 (81%)
Breslow depth	<=1	470 (43.5%)	205 (43.9%)	19 (45.2%)
	1.01-2	18 (1.7%)	4 (0.9%)	2 (4.8%)
	2.01-4	24 (2.2%)	2 (0.4%)	1 (2.4%)
	>4	37 (3.4%)	18 (3.9%)	1 (2.4%)
	Unknown	531 (49.2%)	238 (51%)	19 (45.2%)
Bone metastasis	No	847 (78.4%)	364 (77.9%)	35 (83.3%)
	Yes	233 (21.6%)	103 (22.1%)	7 (16.7%)
Liver metastasis	No	832 (77%)	358 (76.7%)	34 (81%)
	Yes	248 (23%)	109 (23.3%)	8 (19%)
Lung metastasis	No	499 (46.2%)	201 (43%)	20 (47.6%)
	Yes	581 (53.8%)	266 (57%)	22 (52.4%)
Months from diagnosis to therapy	0	672 (62.2%)	280 (60%)	29 (69%)
	>=1	408 (37.8%)	187 (40%)	13 (31%)
Median household income	<$45,000	58 (5.4%)	30 (6.4%)	NA
	$45,000–$59,999	205 (19%)	78 (16.7%)	NA
	$60,000–$74,999	352 (32.6%)	165 (35.3%)	NA
	>$74,999	465 (43.1%)	194 (41.5%)	NA

### 3.2 Univariate and multivariate logistic regression analyses

According to the univariate logistic regression analyses, age, marital status, primary site, radiation, chemotherapy, ulceration, surgery, bone metastasis, liver metastasis, lung metastasis, and months from diagnosis to therapy may be risk factors for the ACED of MBM patients. Age, radiation, chemotherapy, ulceration, surgery, bone metastasis, liver metastasis, lung metastasis, and months from diagnosis to therapy may associated with the CSED of MBM patients.

Multivariate logistic regression analyses revealed that age, marital status, radiation, chemotherapy, ulceration, surgery, bone metastasis, liver metastasis, and months from diagnosis to therapy were significant risk factors for the ACED of MBM patients. Age, radiation, chemotherapy, ulceration, surgery, bone metastasis, liver metastasis, and months from diagnosis to therapy were significant risk factors for the CSED of MBM patients. (**Table 3**)

**Table 3:** Univariate and multivariate logistic regression analyses.

		ACED		CSED	
Variables		Univariable OR (95%CI, P)	Multivariable OR (95%CI, P)	Univariable OR (95%CI, P)	Multivariable OR (95%CI, P)
Age	<40				
	40-65	1.42 (0.82-2.45, p=.206)	1.75 (0.97-3.14, p=.062)	1.47 (0.84-2.55, p=.177)	1.58 (0.88-2.84, p=.126)
	>65	2.56 (1.47-4.43, p<.001)	2.94 (1.61-5.36, p<.001)	2.49 (1.42-4.36, p=.001)	2.38 (1.31-4.33, p=.004)
Sex	Male				
	Female	0.93 (0.71-1.24, p=.633)		0.92 (0.69-1.22, p=.565)	
Race	White				
	Black	2.70 (0.45-16.20, p=.279)		2.88 (0.48-17.31, p=.248)	
	Other	0.90 (0.30-2.65, p=.846)		0.96 (0.33-2.83, p=.941)	
Marital	Married				
	Unmarried	1.31 (1.02-1.69, p=.034)	1.45 (1.10-1.91, p=.008)	1.27 (0.99-1.64, p=.064)	
Primary Site	Skin, NOS				
	Skin other/unspecific parts of face	0.44 (0.12-1.59, p=.209)	0.63 (0.16-2.42, p=.503)	0.29 (0.06-1.29, p=.103)	
	Skin of scalp and neck	0.40 (0.16-1.00, p=.049)	0.39 (0.14-1.07, p=.068)	0.43 (0.17-1.06, p=.068)	
	Skin of trunk	0.71 (0.44-1.15, p=.162)	0.83 (0.46-1.49, p=.532)	0.72 (0.45-1.16, p=.181)	
	Skin of upper limb and shoulder	0.75 (0.38-1.47, p=.397)	1.01 (0.43-2.36, p=.979)	0.80 (0.41-1.56, p=.512)	
	Skin of lower limb and hip	0.43 (0.19-1.01, p=.053)	0.48 (0.19-1.20, p=.115)	0.46 (0.20-1.08, p=.075)	
	Choroid	0.00 (0.00-Inf, p=.987)	0.00 (0.00-Inf, p=.987)	0.00 (0.00-Inf, p=.987)	
	External ear	0.00 (0.00-Inf, p=.978)	0.00 (0.00-Inf, p=.977)	0.00 (0.00-Inf, p=.978)	
	Other rare sites	0.66 (0.27-1.62, p=.367)	0.68 (0.26-1.79, p=.433)	0.57 (0.23-1.46, p=.243)	
Surgery	No				
	Yes	0.55 (0.37-0.82, p=.003)	0.52 (0.29-0.93, p=.026)	0.57 (0.38-0.85, p=.006)	0.47 (0.28-0.77, p=.003)
Radiation	No/Unknown				
	Yes	0.63 (0.46-0.85, p=.002)	0.59 (0.43-0.82, p=.002)	0.68 (0.50-0.92, p=.013)	0.63 (0.46-0.87, p=.005)
Chemotherapy	No/Unknown				
	Yes	0.37 (0.27-0.51, p<.001)	0.39 (0.28-0.54, p<.001)	0.39 (0.28-0.53, p<.001)	0.40 (0.28-0.55, p<.001)
Ulceration	No Ulceration				
	Ulceration	2.15 (1.15-4.04, p=.017)	2.79 (1.30-6.01, p=.008)	2.35 (1.25-4.42, p=.008)	2.78 (1.35-5.74, p=.006)
	Unknown	2.10 (1.47-3.00, p<.001)	1.73 (1.17-2.57, p=.006)	2.14 (1.48-3.08, p<.001)	1.86 (1.26-2.74, p=.002)
Breslow depth	<=1				
	1.01-2	0.38 (0.11-1.33, p=.131)		0.40 (0.11-1.40, p=.151)	
	2.01-4	0.95 (0.40-2.27, p=.909)		1.00 (0.42-2.38, p=.994)	
	>4	1.16 (0.58-2.31, p=.679)		1.21 (0.61-2.42, p=.583)	
	Unknown	1.15 (0.89-1.49, p=.294)		1.10 (0.85-1.43, p=.467)	
Bone metastasis	No				
	Yes	1.68 (1.25-2.26, p<.001)	1.76 (1.24-2.49, p=.001)	1.60 (1.19-2.15, p=.002)	1.56 (1.10-2.19, p=.012)
Liver metastasis	No				
	Yes	1.70 (1.28-2.28, p<.001)	1.69 (1.20-2.39, p=.003)	1.71 (1.28-2.28, p<.001)	1.62 (1.16-2.27, p=.005)
Lung metastasis	No				
	Yes	1.31 (1.02-1.69, p=.033)	1.15 (0.87-1.53, p=.326)	1.38 (1.07-1.78, p=.013)	1.24 (0.94-1.64, p=.135)
Months from diagnosis to therapy	0				
	>=1	0.72 (0.55-0.93, p=.012)	0.60 (0.45-0.80, p<.001)	0.76 (0.59-0.99, p=.045)	0.66 (0.49-0.87, p=.003)
Median household income	<$45,000				
	$45,000–$59,999	1.05 (0.57-1.91, p=.880)		1.01 (0.55-1.83, p=.987)	
	$60,000–$74,999	0.87 (0.49-1.54, p=.629)		0.80 (0.45-1.43, p=.458)	
	>$74,999	0.88 (0.50-1.55, p=.666)		0.82 (0.47-1.44, p=.486)	

### 3.3 Model performance

**Figure 2:**
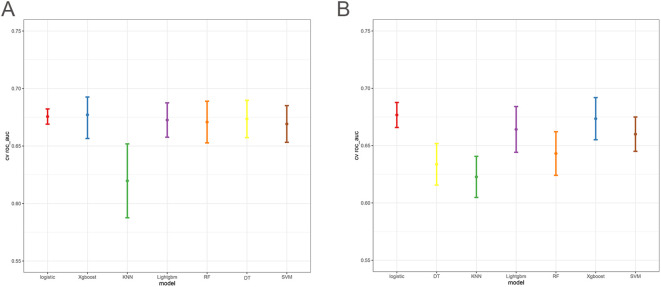
The 10x cross-validation analysis of ACED **(A)**, and CSED **(B)**.

**Figure 3:**
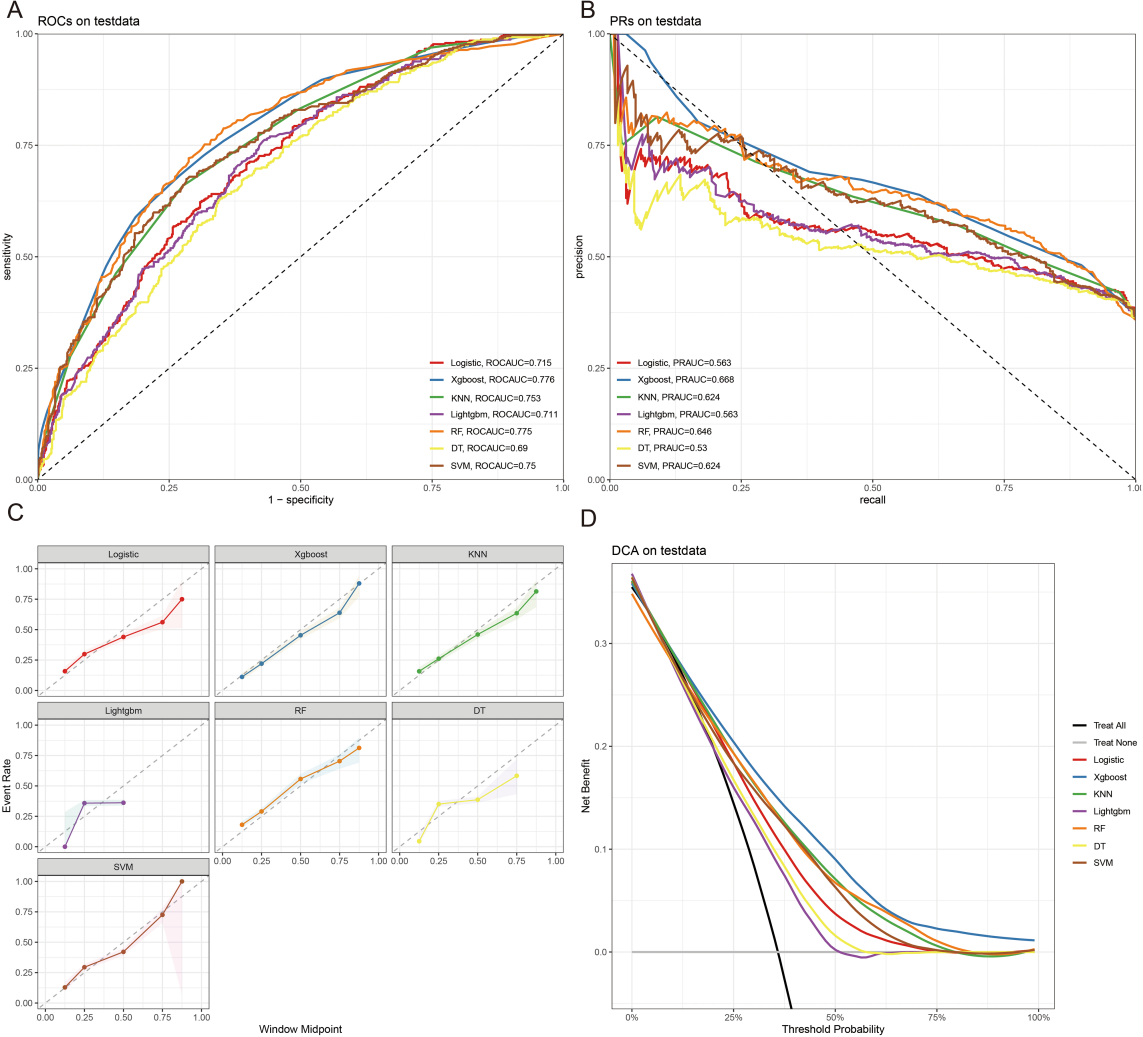
The ROCAUC **(A)**, PRAUC **(B)**, calibration curve **(C)**, and DCA analysis **(D)** of ACED in the test cohort.

**Figure 4:**
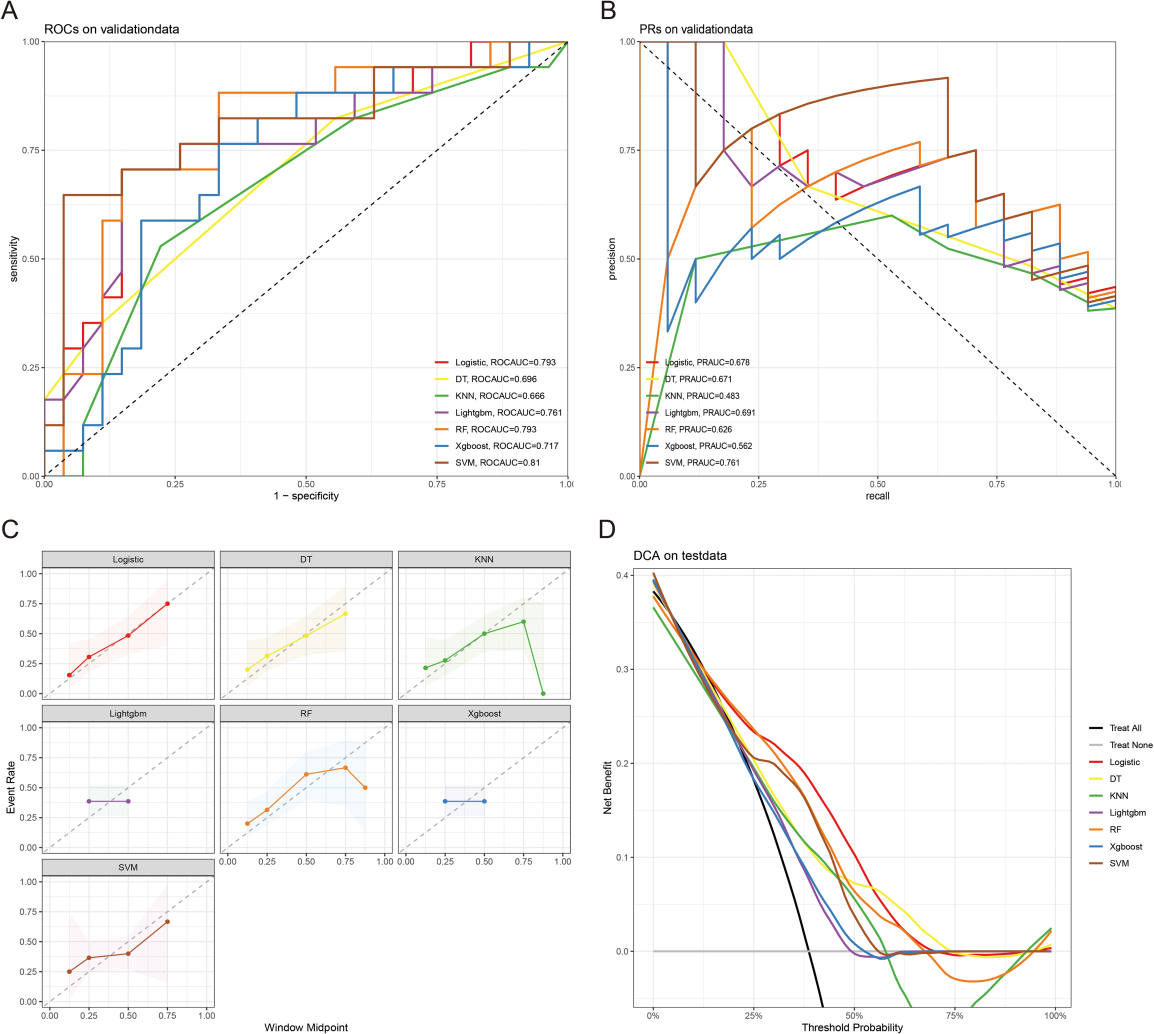
The ROCAUC **(A)**, PRAUC **(B)**, calibration curve **(C)**, and DCA analysis **(D)** of ACED in the external validation cohort.

**Figure 5:**
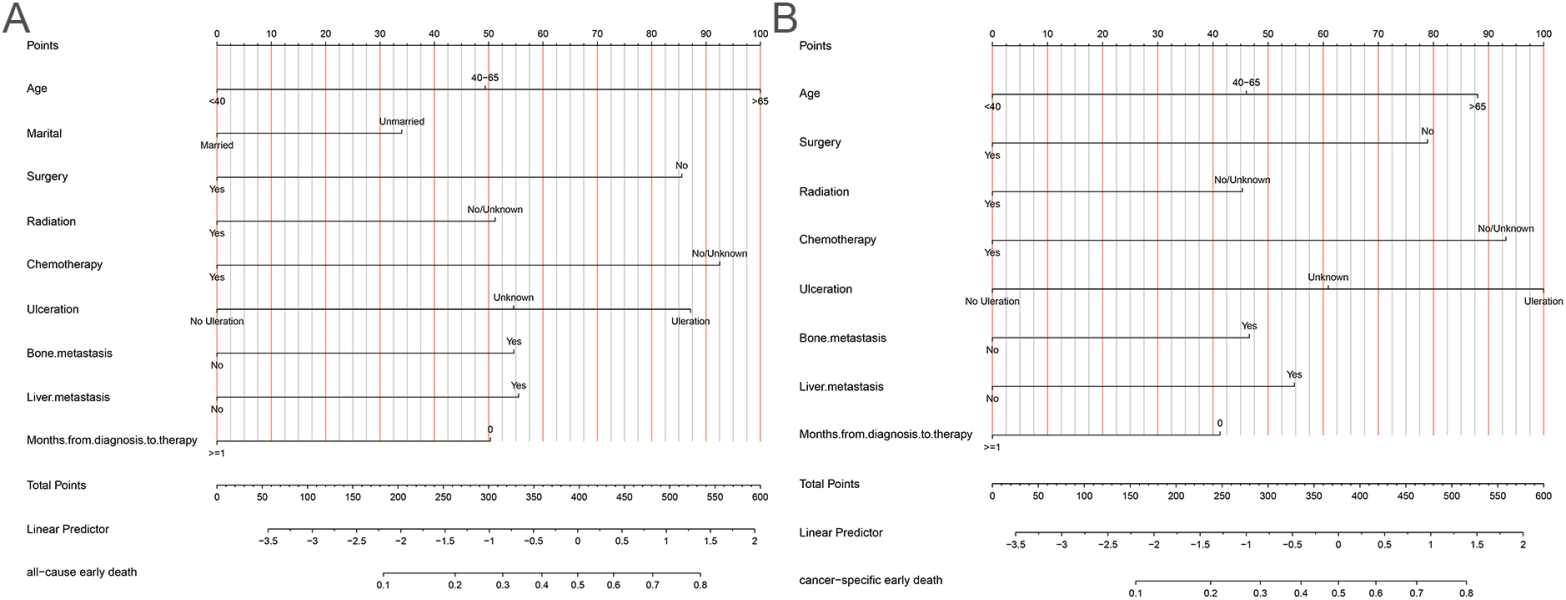
The nomogram of ACED **(A)**, and CSED **(B)**.

The 10-fold cross-validation analysis revealed that the XGBoost model performed best in predicting ACED in MBM patients (**Figure 2A**). In the testing cohort, Xgboost model showed the best with area under curve (AUC)=0.776, accuracy =0.713, sensitivity = 0.682, and specificity = 0.730 (**Figures 3A, B**; **Table 4**). In the external validation cohort, Xgboost model was also shown excellent prediction performance, with AUC=0.717, accuracy =0.659, sensitivity = 0.765, and specificity = 0.593 (**Figures 4A, B**; **Table 4**). Calibration curve analysis revealed that Xgboost model has a more accurate prediction performance (**Figures 3C**, **4C**). DCA curves revealed that Xgboost model clinical application value (**Figures 3D**, **4D**). Besides that, a nomogram based on the Xgboost model showed that age contributes most to the ACED of MBM patients, followed by chemotherapy, ulceration, surgery, liver metastasis, bone metastasis, radiation, months from diagnosis to therapy, and marital status (**Figure 5A**).

**Table 4:** The comparison of model performance.

	ACED				CSED			
Models	AUC	Accuracy	Sensitivity	Specificity	AUC	Accuracy	Sensitivity	Specificity
Test cohort								
Logistic	0.715	0.667	0.623	0.691	0.694	0.614	0.737	0.555
Xgboost	0.776	0.713	0.682	0.730	0.662	0.654	0.524	0.718
KNN	0.753	0.701	0.659	0.724	0.690	0.598	0.729	0.534
Lightgbm	0.711	0.639	0.760	0.571	0.670	0.655	0.529	0.716
RF	0.775	0.697	0.788	0.646	0.691	0.645	0.687	0.624
DT	0.690	0.633	0.672	0.612	0.634	0.514	0.837	0.357
SVM	0.750	0.702	0.674	0.717	0.681	0.674	0.574	0.723
Validation cohort								
Logistic	0.793	0.705	0.765	0.667	0.730	0.659	0.750	0.607
Xgboost	0.717	0.659	0.765	0.593	0.724	0.682	0.750	0.643
KNN	0.666	0.636	0.647	0.630	0.711	0.682	0.750	0.643
Lightgbm	0.761	0.659	0.765	0.593	0.776	0.659	0.813	0.571
RF	0.793	0.705	0.706	0.704	0.739	0.659	0.688	0.643
DT	0.696	0.591	0.824	0.444	0.698	0.568	0.813	0.429
SVM	0.810	0.727	0.824	0.667	0.728	0.682	0.813	0.607

**Figure 6:**
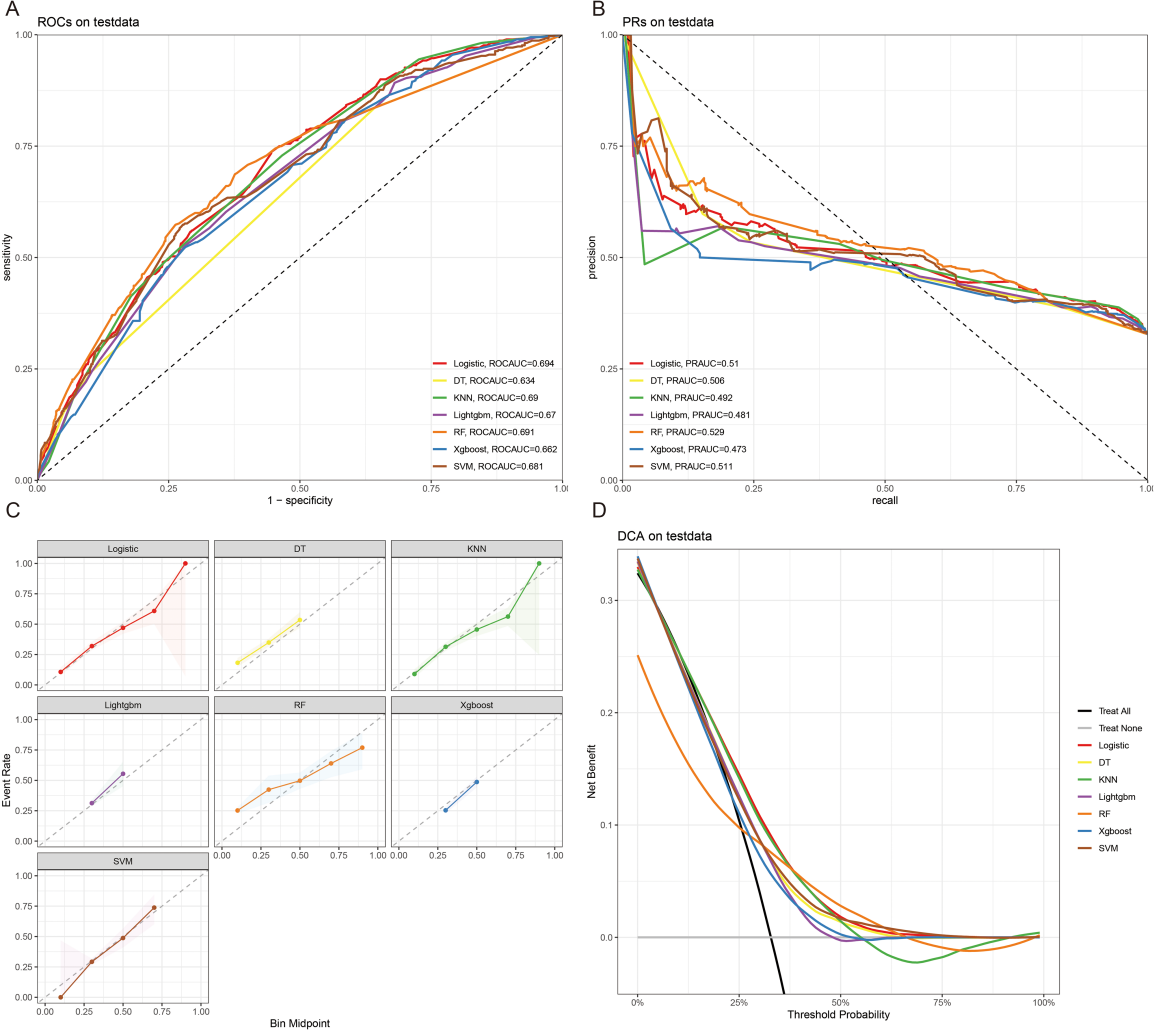
The ROCAUC **(A)**, PRAUC **(B)**, calibration curve **(C)**, and DCA analysis **(D)** of CSED in the test cohort.

**Figure 7:**
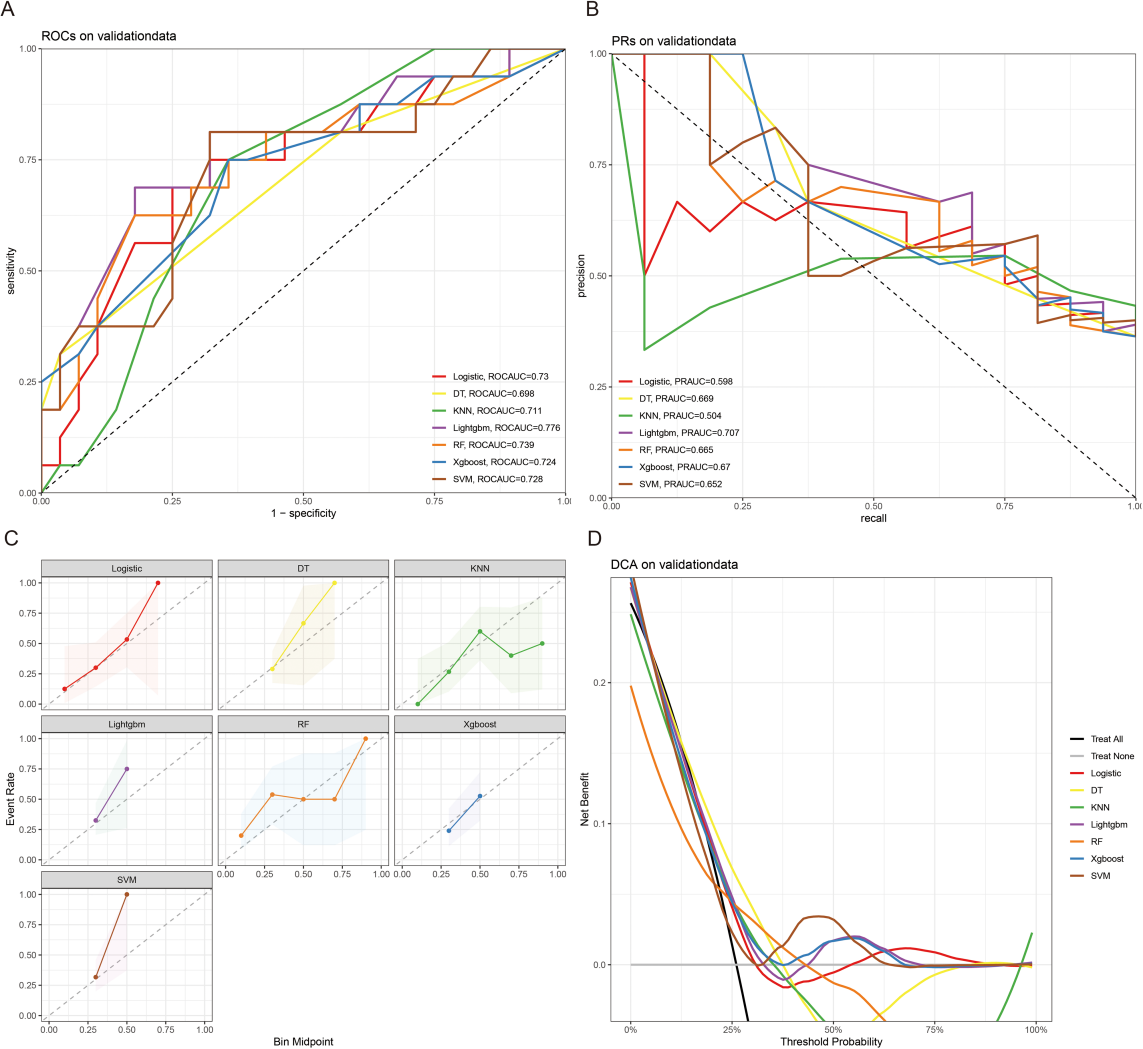
The ROCAUC **(A)**, PRAUC **(B)**, calibration curve **(C)**, and DCA analysis **(D)** of CSED in the external validation cohort.

The 10x cross-validation analysis revealed that the LR model performs best in predicting the CSED of MBM patients (**Figure 2B**). In the testing cohort, the LR model showed the best performance with AUC=0.694, accuracy =0.614, sensitivity = 0.737, and specificity = 0.555 (**Figures 6A, B**; **Table 4**). In the external validation cohort, the LR model also showed excellent prediction performance, with AUC=0.730, accuracy =0.659, sensitivity = 0.750, and specificity = 0.609 (**Figures 7A, B**; **Table 4**). Calibration curve analysis revealed that the LR model has a more accurate prediction performance (**Figures 6C**, **7C**). DCA curves revealed the LR model clinical application value (**Figures 6D**, **7D**). Besides that, a nomogram based on the LR model revealed that ulceration contributes most to the CSED of MBM patients, followed by chemotherapy, age, surgery, liver metastasis, bone metastasis, radiation, and months from diagnosis to therapy (**Figure 5B**).

### 3.4 SHAP analysis results

**Figure 8:**
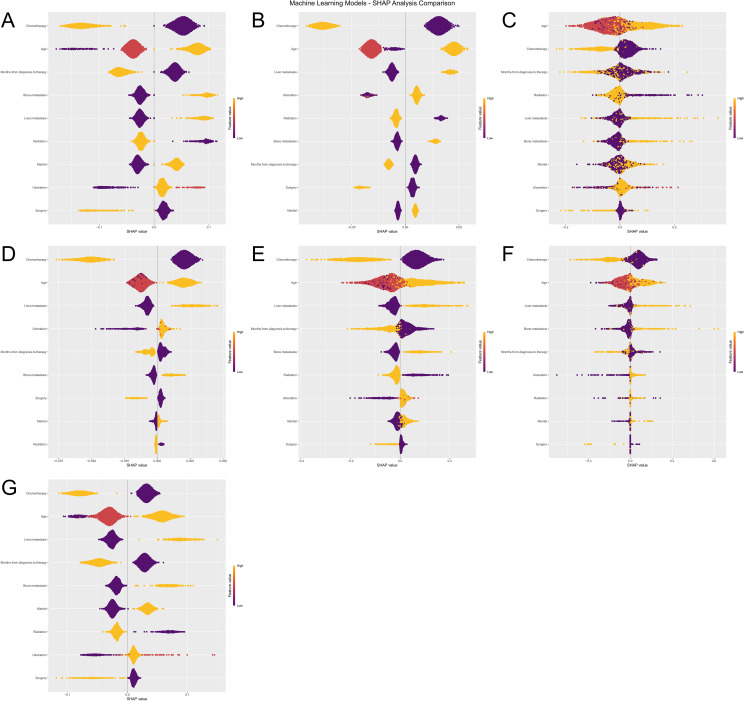
Machine Learning SHAP Analysis Comparison for ACED Outcomes. Subfigures **(A–G)** illustrate the influence of various features (e.g., Age, Chemotherapy, Surgery) on the ACED predictions of seven machine learning models (Logistic Regression, XGBoost, KNN, LightGBM, Random Forest, Decision Tree, and SVM). The violin plots in each subfigure display the distribution of SHAP values for each feature, with color indicating different feature values. Deeper colors represent higher SHAP values, signifying a greater impact of the feature on the prediction results.

**Figure 9:**
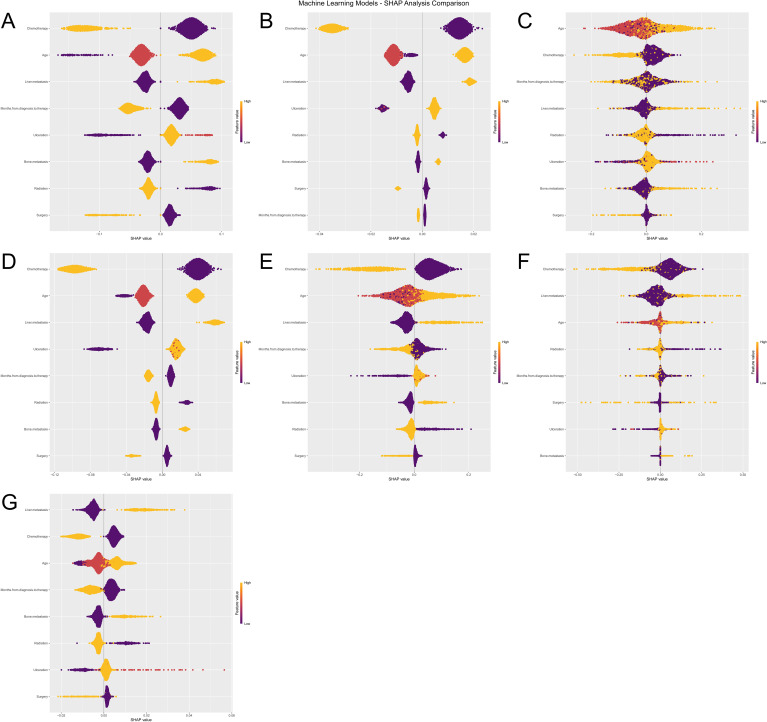
**(A–G)** Machine learning SHAP analysis comparison for CSED outcomes.

To further interpret the machine learning models, we visualized the feature importance using the Shapley Additive Explanations (SHAP) analysis for all seven models predicting both ACED and CSED outcomes (**Figures 8A–G**), (**Figures 9A–G**). In the SHAP plots, variables are arranged in descending order of importance based on their mean absolute SHAP values.

Despite algorithmic differences and different outcome definitions (ACED vs CSED), there was remarkable consistency in feature importance rankings across all seven models (Logistic Regression, XGBoost, KNN, LightGBM, Random Forest, Decision Tree, and SVM). Chemotherapy, age, liver metastasis, and months from diagnosis to therapy consistently emerged as the top four most important features across both ACED and CSED prediction models, highlighting their critical role in predicting early death in melanoma brain metastasis patients regardless of death cause classification.

The consistent ranking of these four variables across different algorithms and outcome definitions suggests their robust prognostic value. The SHAP values provide insights into both the magnitude and direction of each feature’s contribution to the prediction, with higher absolute SHAP values indicating greater influence on the model’s output. This consistency between ACED and CSED models reinforces the reliability of these predictive factors in clinical practice.

## 4 Discussion

Melanoma is a frequent cause of brain metastases, and the survival rate of MBM patients was low (19). Despite the successful development of targeted therapies and immunotherapies that have significantly improved overall survival for MBM patients, the management of MBM remains challenging (20, 21). Brain metastases often lead to poor quality of life (22). Early detection of brain metastases can reduce treatment-related toxicity, mortality, and morbidity (23). In this research, more than a third of patients suffered from ED. Therefore, it is crucial to develop more effective predictive models.

ML is usually used for prognosis prediction and disease diagnosis (24). With the improvement of ML, ML models show great potential in the prediction of treatment and cancer prognosis, refinement of clinical care, and medical research (25). Yi Zhao et al. revealed that combined ML with disulfide ptosis-related signatures showed great significance in predicting the immunotherapy effectiveness and prognosis in cutaneous melanoma (26). Yi Xu et al. identify novel circadian biomarkers for melanoma diagnosis and prognosis through ML approaches (27). In this research, we predicted the ED of MBM patients through clinicopathologic features via ML algorithms, and the AUC value of the Xgboost model of ACED in the test and validation cohort were 0.776, and 0.717 respectively. The AUC values of the LR model of CSED in the test and validation cohort were 0.694, and 0.730 respectively. All AUC values >0.7 indicate that the nomograms have good predictive accuracy. The calibration curve fits the diagonal well. The closer the black solid line is to 45°, the better the prediction effect is and the better the predicted results are in agreement with the actual results. The results show that the predicted early deaths are highly consistent with the actual results. DCA curve revealed that the models have high clinical application value.

Our study presents several noteworthy and novel findings. First, to our knowledge, this study includes the largest cohort of patients, drawing data not only from the SEER database but also from external hospital records. Second, Results revealed that the Xgboost model performs best in predicting the ACED and the LR model performs best in predicting the CSED in MBM patients. In this study, the predictive ML models offered a practical tool for assessing ED risk factors in MBM, aiding physicians in making optimal clinical decisions to improve patient outcomes. ML is being increasingly utilized in clinical settings to assist physicians in making more informed recommendations (28, 29). Compared to traditional data analytics, ML is better suited for handling complex, multi-dimensional big data (30). Notably, lung metastasis was excluded from the final CSED model during backward stepwise regression despite showing univariate significance, likely due to collinearity with other metastatic variables (liver and bone metastases) that demonstrated stronger independent predictive value in the multivariate analysis. Seven ML algorithms were constructed based on the SEER database and hospital data to develop an effective ED prediction tool for MBM patients. Third, in this study, nomograms were developed to quantitatively support individualized prediction of ED based on MBM-related risk factors. Elderly melanoma patients tend to have thicker tumors, a greater likelihood of ulceration, and higher mitotic rates (31). We found that the point of elderly patients in the ACED nomogram is 100, and in the CSED nomogram is about 88, this phenomenon may be linked to the increased sensitivity of the elderly population to the adverse effects of tumor treatment. The presence of ulceration may indicate the relatively rapid progression of melanoma (31). The 5-year survival rate for melanoma significantly decreases in the presence of ulceration (32, 33), and our results showed that patients with ulcers have important significance in promoting ED. Previous studies have shown that radiation, surgery, and chemotherapy are still relatively ideal treatments for MBM, they can reduce the risk of recurrence and prolong the survival of patients (34). However, due to the lack of information on targeted therapies, and immunotherapy in the SEER database, further exploration of treatment options is limited. Given the heterogeneity of MBM patients, accurate risk stratification is crucial, and nomograms offer a convenient tool for this purpose.

This study has several important limitations. First, the information on detailed treatment plans, other treatments, aggregate brain tumor volume, comorbidities, karnofsky performance status that suggest being potential important prognostic factors (35, 36) is unavailable from the SEER database. Additionally, the SEER database does not contain imaging data, precluding the inclusion of important radiological features such as tumor size, shape, location, edema, and enhancement patterns. These imaging characteristics could potentially enhance model performance, as demonstrated in previous studies. Second, this retrospective analysis did not utilize real clinical external data for prospective validation, which will need to be addressed in future research. Third, our dataset is geographically limited to SEER populations (representing selected US regions) and a single regional Chinese hospital, which may limit the generalizability of our findings to other global populations. Population genetics, tumor biology, healthcare system differences, and socioeconomic factors may vary significantly across different geographical regions, potentially affecting model performance and clinical applicability. While our two-population validation using both Western and Asian cohorts demonstrates some cross-population validity, broader validation across diverse geographical regions, healthcare systems, and ethnic populations is essential before widespread clinical implementation. Fourth, our external validation cohort from the Affiliated Cancer Hospital of Xinjiang Medical University was relatively small (n=42) compared to the training and testing cohorts, which may limit the robustness of our generalizability assessment. While this sample size reflects the relative rarity of MBM cases at a single institution and still demonstrated consistent model performance, larger multi-institutional external validation studies are needed to more definitively establish model reliability across diverse clinical settings. Future studies should prioritize multi-continental validation and consider population-specific model recalibration to optimize performance for local clinical use.

## 5 Conclusion

Seven ML algorithms were used to establish ED predictive models of MBM patients by demographic characteristics. Xgboost model exhibited the best predictive ability for ACED, and LR model performs best in predicting the CSED, indicating potential clinical application value.

## Data Availability

The datasets presented in this study can be found in online repositories. The names of the repository/repositories and accession number(s) can be found in the article/supplementary material.

## References

[1] Gutzmer R Vordermark D Hassel JC Krex D Wendl C SChadendorf D. Melanoma brain metastases - Interdisciplinary management recommendations 2020. Cancer Treat Rev. (2020) 89:102083. doi: 10.1016/j.ctrv.2020.102083, PMID: 32736188

[2] Conway JW Rawson RV Lo S Ahmed T Vergara IA Gide TN. Unveiling the tumor immune microenvironment of organ-specific melanoma metastatic sites. J Immunother Cancer. (2022) 10(9):e004884. doi: 10.1136/jitc-2022-004884, PMID: 36096531 PMC9472156

[3] Ascha MS Ostrom QT Wright J Kumthekar P Bordeaux JS Sloan AE. Lifetime occurrence of brain metastases arising from lung, breast, and skin cancers in the elderly: A SEER-medicare study. Cancer Epidemiol Biomarkers Prev. (2019) 28:917–25. doi: 10.1158/1055-9965.EPI-18-1116, PMID: 31053636 PMC6506177

[4] Cohen JV Tawbi H Margolin KA Amravadi R Bosenberg M Brastianos PK. Melanoma central nervous system metastases: current approaches, challenges, and opportunities. Pigment Cell Melanoma Res. (2016) 29:627–42. doi: 10.1111/pcmr.12538, PMID: 27615400 PMC5398760

[5] Sadetsky N Hernandez A Wallick CJ McKenna EF Surinach A Colburn DE. Survival outcomes in an older US population with advanced melanoma and central nervous system metastases: SEER-Medicare analysis. Cancer Med. (2020) 9:6216–24. doi: 10.1002/cam4.3256, PMID: 32667719 PMC7476818

[6] Liu H Xu YB Guo CC Li MX Ji JL Dong RR. Predictive value of a nomogram for melanomas with brain metastases at initial diagnosis. Cancer Med. (2019) 8:7577–85. doi: 10.1002/cam4.2644, PMID: 31657530 PMC6912053

[7] Sundararajan S Thida AM Yadlapati S Mukkamalla SKR Koya S. Metastatic melanoma. In: StatPearls. StatPearls Publishing LLC, Treasure Island (FL (2024). 29262232

[8] Zhu Y Fang X Wang L Zhang T Yu D. A predictive nomogram for early death of metastatic gastric cancer: A retrospective study in the SEER database and China. J Cancer. (2020) 11:5527–35. doi: 10.7150/jca.46563, PMID: 32742500 PMC7391207

[9] Shen H Deng G Chen Q Qian J. The incidence, risk factors and predictive nomograms for early death of lung cancer with synchronous brain metastasis: a retrospective study in the SEER database. BMC Cancer. (2021) 21:825. doi: 10.1186/s12885-021-08490-4, PMID: 34271858 PMC8285786

[10] Koch Hein EC Vilbert M Hirsch I. Immune checkpoint inhibitors in advanced cutaneous squamous cell carcinoma: real-world experience from a Canadian Comprehensive Cancer Centre. Cancers (Basel). (2023) 15(17):4312. doi: 10.3390/cancers15174312, PMID: 37686588 PMC10487051

[11] Nieder C Aanes SG Haukland EC. Survival and early death within three months from the start of immune checkpoint inhibitors in patients with different types of cancer. Anticancer Res. (2022) 42:3061–6. doi: 10.21873/anticanres.15793, PMID: 35641252

[12] Li S Yin C Yang X Lu Y Wang C Liu B. Risk factors and predictive models for early death in patients with advanced melanoma: A population-based study. Med (Baltimore). (2023) 102:e35380. doi: 10.1097/MD.0000000000035380, PMID: 37800813 PMC10552983

[13] Li S Li J Yang Q Yin C Liu B. Construction and validation of prediction models of risk factors for early death in patients with metastatic melanoma. Sichuan Da Xue Xue Bao Yi Xue Ban. (2024) 55:367–74. doi: 10.12182/20240360101, PMID: 38645854 PMC11026897

[14] Zhang D Wang Z Shang D Yu J Yuan S. Incidence and prognosis of brain metastases in cutaneous melanoma patients: a population-based study. Melanoma Res. (2019) 29:77–84. doi: 10.1097/CMR.0000000000000538, PMID: 30379726

[15] Zhou SN Jv DW Meng XF Zhang JJ Liu C Wu ZY. Feasibility of machine learning-based modeling and prediction using multiple centers data to assess intrahepatic cholangiocarcinoma outcomes. Ann Med. (2023) 55:215–23. doi: 10.1080/07853890.2022.2160008, PMID: 36576390 PMC9809369

[16] Ainiwaer A Hou WQ Qi Q Kadier K Qin L Rehemuding R. Deep learning of heart-sound signals for efficient prediction of obstructive coronary artery disease. Heliyon. (2024) 10:e23354. doi: 10.1016/j.heliyon.2023.e23354, PMID: 38169906 PMC10758826

[17] Lynch CM Abdollahi B Fuqua JD de Carlo AR Bartholomai JA Balgemann RN. Prediction of lung cancer patient survival via supervised machine learning classification techniques. Int J Med Inform. (2017) 108:1–8. doi: 10.1016/j.ijmedinf.2017.09.013, PMID: 29132615 PMC5726571

[18] Zhang Y Zhang Z Wei L Wei S. Construction and validation of nomograms combined with novel machine learning algorithms to predict early death of patients with metastatic colorectal cancer. Front Public Health. (2022) 10:1008137. doi: 10.3389/fpubh.2022.1008137, PMID: 36605237 PMC9810140

[19] Switzer B Puzanov I Skitzki JJ Hamad L Ernstoff MS. Managing metastatic melanoma in 2022: A clinical review. JCO Oncol Pract. (2022) 18:335–51. doi: 10.1200/OP.21.00686, PMID: 35133862 PMC9810138

[20] Sperduto PW Salama AKS Anders C. Progress for patients with melanoma brain metastases. Neuro Oncol. (2023) 25:1321–2. doi: 10.1093/neuonc/noad050, PMID: 36883201 PMC10326485

[21] Fateeva A Eddy K Chen S. Overview of current melanoma therapies. Pigment Cell Melanoma Res. (2023) 37(5):562–8. doi: 10.1111/pcmr.13154, PMID: 38063139 PMC11161550

[22] Brown PD Jaeckle K Ballman KV Farace E Cerhan JH Anderson SK. Effect of radiosurgery alone vs radiosurgery with whole brain radiation therapy on cognitive function in patients with 1 to 3 brain metastases: A randomized clinical trial. Jama. (2016) 316:401–9. doi: 10.1001/jama.2016.9839, PMID: 27458945 PMC5313044

[23] Chang EL Wefel JS Hess KR Allen PK Lang FF Kornguth DG. Neurocognition in patients with brain metastases treated with radiosurgery or radiosurgery plus whole-brain irradiation: a randomised controlled trial. Lancet Oncol. (2009) 10:1037–44. doi: 10.1016/S1470-2045(09)70263-3, PMID: 19801201

[24] Handelman GS Kok HK Chandra RV Razavi AH Lee MJ Asadi H. eDoctor: machine learning and the future of medicine. J Intern Med. (2018) 284:603–19. doi: 10.1111/joim.12822, PMID: 30102808

[25] Li J Dan K Ai J. Machine learning in the prediction of immunotherapy response and prognosis of melanoma: a systematic review and meta-analysis. Front Immunol. (2024) 15:1281940. doi: 10.3389/fimmu.2024.1281940, PMID: 38835779 PMC11148209

[26] Zhao Y Wei Y Fan L Nie Y Li J Zeng R. Leveraging a disulfidptosis-related signature to predict the prognosis and immunotherapy effectiveness of cutaneous melanoma based on machine learning. Mol Med. (2023) 29:145. doi: 10.1186/s10020-023-00739-x, PMID: 37884883 PMC10601311

[27] Xu Y Zeng C Bin J Tang H Li W. Identifying novel circadian rhythm biomarkers for diagnosis and prognosis of melanoma by an integrated bioinformatics and machine learning approach. Aging (Albany NY). (2024) 16:11824–42. doi: 10.18632/aging.205961, PMID: 39213172 PMC11386929

[28] Miao R Chen HH Dang Q Xia LY Yang ZY He MF. Beyond the limitation of targeted therapy: Improve the application of targeted drugs combining genomic data with machine learning. Pharmacol Res. (2020) 159:104932. doi: 10.1016/j.phrs.2020.104932, PMID: 32473309

[29] Mosele F Remon J Mateo J Westphalen CB Barlesi F Lolkema MP. Recommendations for the use of next-generation sequencing (NGS) for patients with metastatic cancers: a report from the ESMO Precision Medicine Working Group. Ann Oncol. (2020) 31:1491–505. doi: 10.1016/j.annonc.2020.07.014, PMID: 32853681

[30] Deo RC. Machine learning in medicine. Circulation. (2015) 132:1920–30. doi: 10.1161/CIRCULATIONAHA.115.001593, PMID: 26572668 PMC5831252

[31] Shen W Sakamoto N Yang L. Melanoma-specific mortality and competing mortality in patients with non-metastatic Malignant melanoma: a population-based analysis. BMC Cancer. (2016) 16:413. doi: 10.1186/s12885-016-2438-3, PMID: 27389173 PMC4936003

[32] Huang JN Yu H Wan Y Ming WK Situ F Zhu L. A prognostic nomogram for the cancer-specific survival of white patients with invasive melanoma at BANS sites based on the Surveillance, Epidemiology, and End Results database. Front Med (Lausanne). (2023) 10:1167742. doi: 10.3389/fmed.2023.1167742, PMID: 37497274 PMC10366473

[33] Maurichi A Miceli R Camerini T Mariani L Patuzzo R Ruggeri R. Prediction of survival in patients with thin melanoma: results from a multi-institution study. J Clin Oncol. (2014) 32:2479–85. doi: 10.1200/JCO.2013.54.2340, PMID: 25002727

[34] Gao M Wu B Bai X. Establishment and validation of a nomogram model for predicting the specific mortality risk of melanoma in upper limbs based on the SEER database. Sci Rep. (2024) 14:9623. doi: 10.1038/s41598-024-57541-w, PMID: 38671023 PMC11053139

[35] Hauswald H Stenke A Debus J Combs SE. Linear accelerator-based stereotactic radiosurgery in 140 brain metastases from Malignant melanoma. BMC Cancer. (2015) 15:537. doi: 10.1186/s12885-015-1517-1, PMID: 26201853 PMC4511446

[36] Shultz DB Modlin LA Jayachandran P Von Eyben R Gibbs IC Choi CYH. Repeat courses of stereotactic radiosurgery (SRS), deferring whole-brain irradiation, for new brain metastases after initial SRS. Int J Radiat Oncol Biol Phys. (2015) 92:993–9. doi: 10.1016/j.ijrobp.2015.04.036, PMID: 26194677

